# The ankle in XLH: Reduced motion, power and quality of life

**DOI:** 10.3389/fendo.2023.1111104

**Published:** 2023-03-22

**Authors:** Celine Akta, Florian Wenzel-Schwarz, Alexandra Stauffer, Andreas Kranzl, Adalbert Raimann, Roland Kocijan, Rudolf Ganger, Gabriel T. Mindler

**Affiliations:** ^1^ Department of Pediatric Orthopaedics, Orthopaedic Hospital Speising, Vienna, Austria; ^2^ Vienna Bone and Growth Center, Vienna, Austria; ^3^ Medical Faculty of Bone Diseases, Sigmund Freud University, Vienna, Austria; ^4^ Laboratory for Gait and Movement Analysis, Orthopaedic Hospital Speising, Vienna, Austria; ^5^ Comprehensive Center for Pediatrics, Department of Pediatrics and Adolescent Medicine, Division of Pediatric Pulmonology, Allergology and Endocrinology, Medical University of Vienna, Vienna, Austria; ^6^ 1st Medical Department, Ludwig Boltzmann Institute of Osteology at Hanusch Hospital of OEGK and AUVA Trauma Centre Meidling, Hanusch Hospital, Vienna, Austria

**Keywords:** deformity, gait analysis, enthesopathy, XLH, hypophosphatemia, osteoarthritis, ankle, ankle power

## Abstract

**Background:**

X-linked hypophosphatemia (OMIM 307800) is a rare bone disease caused by a phosphate-wasting condition with lifelong clinical consequences. Those affected suffer from bone pain, complex skeletal deformities, impaired mobility and a reduced quality of life. Early osteoarthritis and reduced range of motion of the lower limbs are known pathologies in XLH patients. However, XLH-specific data on the affected compartments such as the ankle joint through the evaluation of radiographic and gait analysis data is still lacking.

**Patients and methods:**

In this cross-sectional study, patients with genetically verified XLH, age ≥ 16 - 50 years and a complete record of gait analysis and or radiographic analysis data were included. Clinical examination, radiological and gait analysis data were compared to norms using the dataset of our gait laboratory registry. Radiographic analysis included tibial deformity analysis and assessment of osteoarthritis and enthesopathies. Western Ontario and McMaster Universities Arthritis Index (WOMAC), SF36v2, American Orthopedic Foot and Ankle Society score (AOFAS) and the Foot and Ankle Outcome Score (FAOS) were used. Twentythree participants with 46 limbs were eligible for the study.

**Results:**

A total of 23 patients (n=46 feet) met the inclusion criteria. Patients with XLH had significantly reduced gait quality, ankle power and plantar flexion (p < 0.001) compared to a historic gait laboratory control group. Ankle valgus deformity was detected in 22 % and ankle varus deformity in 30 % of the patients. The subtalar joint (59.1%) as well as the anterior tibiotalar joint (31.1%) were the main localizations of moderate to severe joint space narrowing. Ankle power was decreased in moderate and severe subtalar joint space narrowing (p < 0.05) compared to normal subtalar joint space narrowing. No lateral or medial ligament instability of the ankle joint was found in clinical examination. Tibial procurvatum deformity led to lower ankle power (p < 0.05).

**Conclusions:**

This study showed structural and functional changes of the ankle in patients with XLH. Subtalar ankle osteoarthritis, patient reported outcome scores and clinical ankle restriction resulted in lower gait quality and ankle power.

## Introduction

X-linked hypophosphatemia (XLH, OMIM 307800) is the most common type of hereditary hypophosphatemia. It is caused by a loss-of-function mutation in the PHEX gene (phosphate-regulating-gene with homology to endopeptidases on the X-chromosome) causing renal phosphate loss and impairment of 1,25 vitamin D due to the high levels of fibroblast growth factor 23 (FGF-23) (1).

Most commonly, XLH is diagnosed in childhood and treatment is conducted by multidisciplinary specialist care. Nevertheless, XLH has lifelong clinical consequences such as short stature, musculoskeletal pain, complex skeletal deformities, disability among adults, premature osteoarthritis (OA) and enthesopathies (1–3). Lower limb deformities frequently occur in children and adolescents with XLH (4, 5) possibly caused by changes in bone growth and/or surgical interventions (4). The skeletal deformities lead to impaired gait and decreased quality of life (QoL), which are associated with a high burden of disease in XLH patients (1–3). It has been reported that more than 57% of adults with XLH have a history of orthopedic surgeries, even though the patients had received conventional supplementation therapy with active vitamin D and/or phosphates metabolites or analogues in their childhood (3).

Furthermore, functional impairments such as gait abnormalities (2–4, 6), reduced joint motion (4, 6) as well as fatigue (3) have been recently described in adults with XLH.

A recent study reported that 97% of adults and 80% of children with XLH described bone or joint pain and or stiffness, as well as 51% of adults and 21% of children reported ankle joint pain (2). XLH is known for early OA development (1, 3, 4), not only affecting the hip and knee joints, but also present in the especially high prevalence of ankle OA (4). The human foot is part of a biomechanical chain depending on other limbs. Impaired biomechanical function, thus, impaired range of motion of the ankle and foot, overloads adjacent joints and disturbs the gait pattern. OA can impair ROM and represents a process of cartilage degeneration, which makes normal articular joint loading essential to maintaining a functional joint (7). Disturbances of biomechanical function, such as obesity and lower limb deformity, which was associated with low gait quality scores in adults (4) and children (5) with XLH, could lead to joint overload, thus possibly contributing to early OA development.

This study was a follow up of our previous study (4) on lower limb deformity in XLH patients, after ankle problems were identified. Although XLH research is progressing, it has not yet focused on the impact of physical manifestations of XLH on functional range of motion and mobility of the ankle as a crucial joint for lower limb mobility. This study aims to appraise the impact of the disease on the ankle function of adolescent and adult patients with XLH through the evaluation of clinical, radiological, and gait analysis data.

Our main objective was to evaluate ankle function in adolescents and adults with XLH and compare it to a historic healthy control group. Primary outcome parameters included ankle ROM in clinical examination and gait analysis as well as radiologic parameters for deformity and osteoarthritis.

The secondary objective was to analyze whether ankle impairment decreases quality of life parameters in adolescents and adults with XLH assessed by obtaining questionnaire and functional scores through the Western Ontario and McMaster Universities Arthritis Index (WOMAC), SF36v2, American Orthopedic Foot and Ankle Society score (AOFAS) and the Foot and Ankle Outcome Score (FAOS).

The assessment of scores, clinical and radiological data as well as gait analysis was performed to test the hypothesis of this study that the ankle shows structural and functional impairments in adolescents and adults with XLH, which could also impact quality of life in this rare bone disease.

## Patients and methods

This cross-sectional single-center study including adolescent and adult patients with XLH performed at the Orthopedic Hospital Speising, Vienna (part of the Vienna Bone and Growth Center), obtained and compared data to norms using the dataset of our gait laboratory registry. We retrospectively analyzed gait analysis and radiographic data which were routinely obtained in adolescents and adults with XLH. Furthermore, patients were actively recruited *via* the Austrian XLH patient organization to participate in this study with a one-time gait analysis, radiographic evaluation and foot/QoL scores.

The study was approved by the Ethics Committee of the Vinzenz Group Vienna (EK 37/2020).

The inclusion criteria consisted of patients aged between 16 and 50 years with genetically verified XLH, as well as a complete record of gait analysis and/or radiographic analysis data.

Pregnant patients and patients with other types of hypophosphatemia were excluded. Patients with a recent (within 12 months) foot or ankle trauma were excluded. The upper age limit of 50 years was chosen considering the prevalence for abnormal gait in elderly patients above 70 years is estimated to be 35% (8) and the prevalence of osteoarthritis is predicted to affect 9.6% of men and 18% of women aged >60 years (9). Laboratory examination and gait analysis results within one year of radiographic and clinical examination were included in the analysis. According to the World Health Organization a body mass index (BMI) over or equal to 25 was defined as overweight, a BMI over or equal to 30 as obese (10).

During the period from April 2018 to September 2021, data on 26 adolescent and adult patients was collected for the study. Three patients were excluded for exceeding the age limit of 50 years. Therefore, 23 participants with 46 limbs were eligible, eighteen of which (78.3%) were female, 8 participants were aged 16 - 24 years and 15 were aged 25 - 50 years.

**Figure 1:**
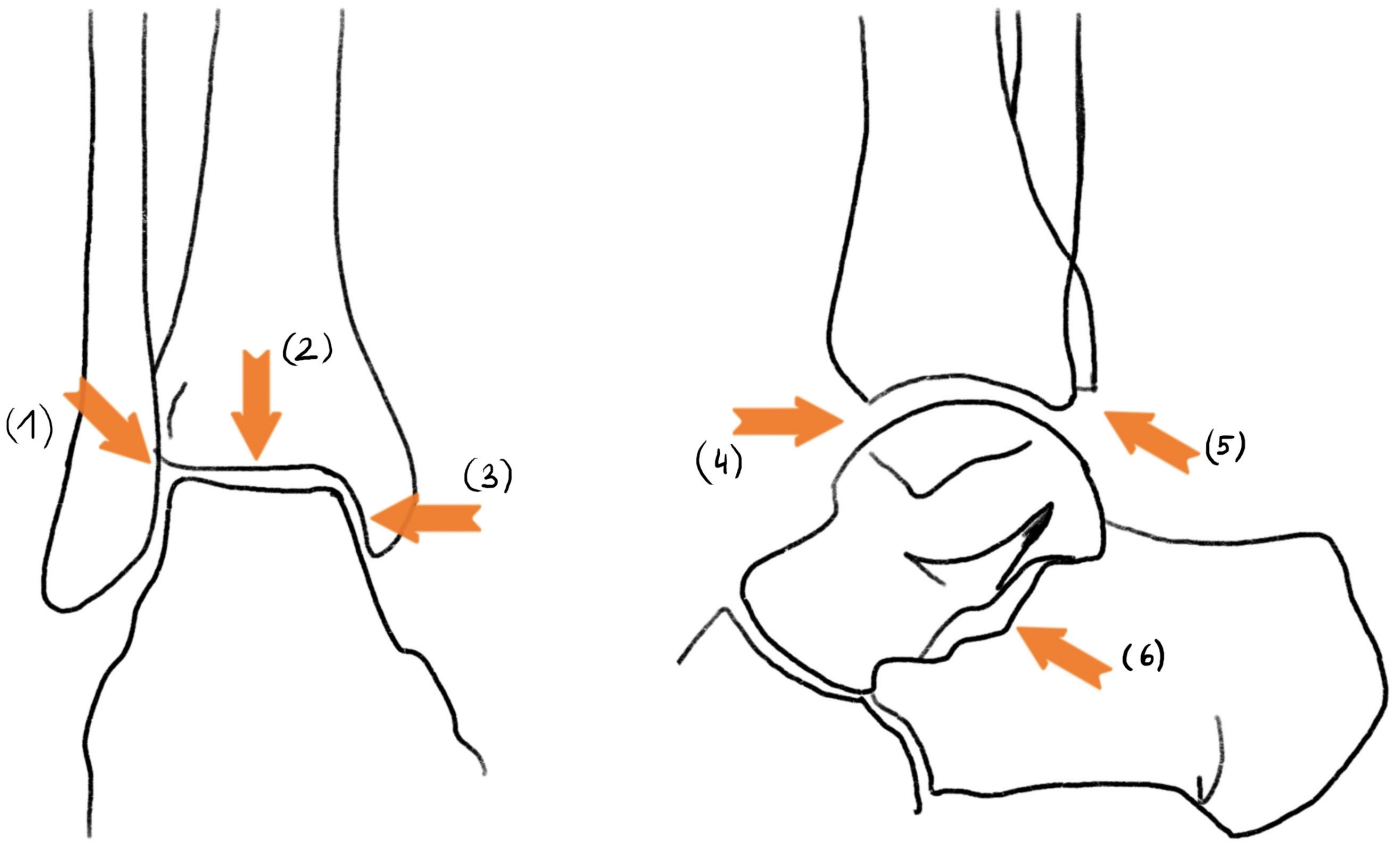
JSN measuring in reference to Kraus et al.: (1): talofibular JSN; (2): superior tibiotalar JSN; (3): medial tibiotalar JSN; (4): anterior tibiotalar JSN; (5): posterior tibiotalar JSN; (6): subtalar JSN.

Anteroposterior (ap) and lateral radiographs of both ankles and tibiae in standing position with a calibration ball were obtained to analyze deformity, joint space narrowing (JSN) and enthesopathy presence. Inadequately displayed joints on X-rays (due to malrotation) were excluded for each sub analysis if necessary. Deformity analysis of ap and lateral radiograph images of the tibia was performed by one examiner (A.S) according to standard measurements (Lateral distal tibial angle (LDTA), medial proximal tibial angle, proximal posterior tibial angle, anterior distal tibial angle) (11). Normal ankle alignment was defined as LDTA between 86 and 92 degrees, Varus malalignment was defined as LDTA > 92°, valgus malalignment as LDTA < 86° (11). JSN and osteophytes of the ankle were graded according to Kraus et al. (12) and was performed by a senior foot and ankle surgeon (F.W-S.). In this study, OA refers to the joint occurrence of JSN and osteophytes. Three joints were included in grading: the tibiotalar-, talofibular- and subtalar joint. JSN was graded “normal”, “mild”, “moderate” and “severe”. Osteophytes were graded “absent”, “small”, “moderate”, “large”. Results were presented for osteophytes and JSN for gradings mild to severe if not stated otherwise (**Figures 1**, **2** in the supplements section illustrate the exact measuring locations).

**Figure 2:**
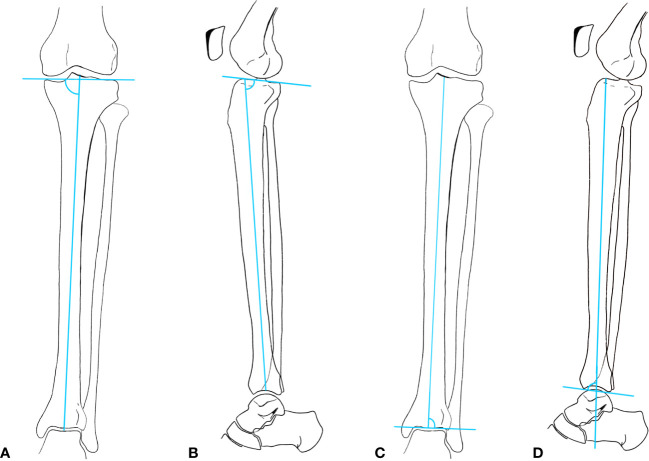
**(A)** medial proximal tibial angle (MPTA); **(B)** Proximal posterior tibial angle (PPTA); **(C)** Lateral distal tibial angle (LDTA); **(D)** Anterior distal tibial angle (ADTA).

Gait analysis using a modified Cleveland model for movement of the lower extremity and a Plug in Gait model for movement of the upper extremity marker set was conducted (13, 14) at the laboratory for gait and movement analysis at the Orthopedic Hospital Speising, Vienna. Patients walked a 12-meter walkway barefoot at a self-selected speed. Kinetic data were calculated from a minimum of five force plate strikes (force plates: AMTI Advanced Mechanical Technology Inc., Watertown, Massachusetts) per foot. Kinematic data were collected from a minimum of 10 gait cycles. A custom Matlab script (The MathWorks, Natick, Massachusetts, Version 2019a) was used for graphing of data and comparisons between groups. The Gait Deviation Index (GDI) was calculated according to Schwartz and Rozumalski (15). Kinematic measurements of the trunk, pelvis, hips, knees and ankles were recorded as joint angles in the sagittal, frontal and transverse planes. The gait cycle was mainly analyzed in the sagittal plane, focusing on the dorsiflexion and plantarflexion of the ankle. Additionally, kinetic parameters such as ankle power and moment were measured. Maximal ankle power was measured at push off with AMTI force plates (Advanced Mechanical Technology, Inc., Watertown, MA) which are embedded in the ground.

### Statistics

The statistical analysis was performed using SPSS (IBM SPSS Statistics, version 27). The standard distribution was reviewed using the Kolmogorov-Smirnov-Test, then a single sample t-test was used to evaluate statistically significant results in comparison to norm values (alpha level was set at p < 0.05). An independent-samples t-test and one-way analysis of variance were used, respectively, to quantify the difference of gait parameter changes on clinical, radiological and self-reported questionnaires in variables with two or more groups. *Post-hoc* analysis was performed using TukeyHSD (Tukey’s Honestly-Significant Difference) *post-hoc* test to evaluate which differences in the groups were significant.

Levene’s test, normality checks and homogeneity tests were carried out and the assumptions met. For parameters with normal and non-normal distribution, Pearson’s or Spearman’s correlations were calculated, respectively. Data with normal distribution were assessed using the Shapiro–Wilk test. Statistical parametric mapping was used to assess waveforms (16). The statistics of gait parameters were calculated at Matlab (2020b, Mathworks) and SPM in Python (Statistical Parametric Mapping. Retrieved from www.spm1d.org).

## Results

### Study population and demographics

XLH patients showed decreased standing height and a higher frequency of pre-obesity and obese BMI compared to the average Austrian population (17). Ten patients (10 of 17, 58.8%) measured a BMI over 25, out of which 35.3% were pre-obese (BMI: > 25 kg/m^2^) and 23% were defined as obese (BMI: > 30 kg/m^2^).

Laboratory findings (n = 13) showed alkaline phosphatase (ALP) and calcium values within the upper normal range and calcidiol levels within the normal range; decreased phosphate levels and elevated parathyroid hormone (PTH) values (**Table 1**). We did not find a significant correlation between laboratory values and radiographic or gait analysis data. Phosphate, vitamin D and calcium therapy was actively taken by 9 XLH patients. No patient underwent Burosumab therapy at that time.

**Table 1:** Characteristics and laboratory findings of included XLH patients.

	N	Mean	SD	Min	Max	Reference Range
Age at diagnosis (years)	23	32.9	11.0	16	50	
Weight (kg)	17	67.8	15.3	48.0	96.4	
Height (cm)	17	155.7	7.9	141.0	169.0	
BMI (kg/m^2^)	17	28.1	7.1	18.4	42.2	
Calcium (mmol/l)	13	2.4	0.2	2.1	2.6	2.3 – 2.6
Phosphate (mmol/l)	13	0.7	0.2	0.4	1.2	0.77 – 1.55
ALP (U/l)	11	124.3	47.1	69.0	202.0	65 – 220
25 OH-D (nmol/l)	13	46.7	24.5	20.5	98.0	37 – 200
PTH (ng/L)	13	86.6	55.1	28.4	202.0	10 – 65
Active plantar flexion (deg)	20 (l)	45.2	12.2	20	60	45 - 50 *
Active dorsiflexion (deg)	20 (l)	16.7	8.6	0	32	20 - 30 *
Weight bearing lunge test (WBLT; cm)	18 (l)	6.1	4.4	0	14.5	12 ± 2.8 **
AOFAS: Hindfoot	37 (l)	86.1	16.7	48	100	
AOFAS: Midfoot	37 (l)	90.5	14.8	43	100	
QoL (FAOS)	35 (l)	58.3	21.1	10	80	

*SD, Standard deviation; BMI, Body mass index; kgBW, kg body weight; U, Units; deg, degrees; (l), limbs; * (7); ** (14).

XLH patients reported to have undergone bony surgery an average 5.65 times per person (± 4.9; range: 0 to 17). Two patients reported to be surgery naïve.

### Radiographic results

All patients (n = 23) had a complete radiographic record of both limbs (n = 46).

### Deformity analysis

**Figure 3:**
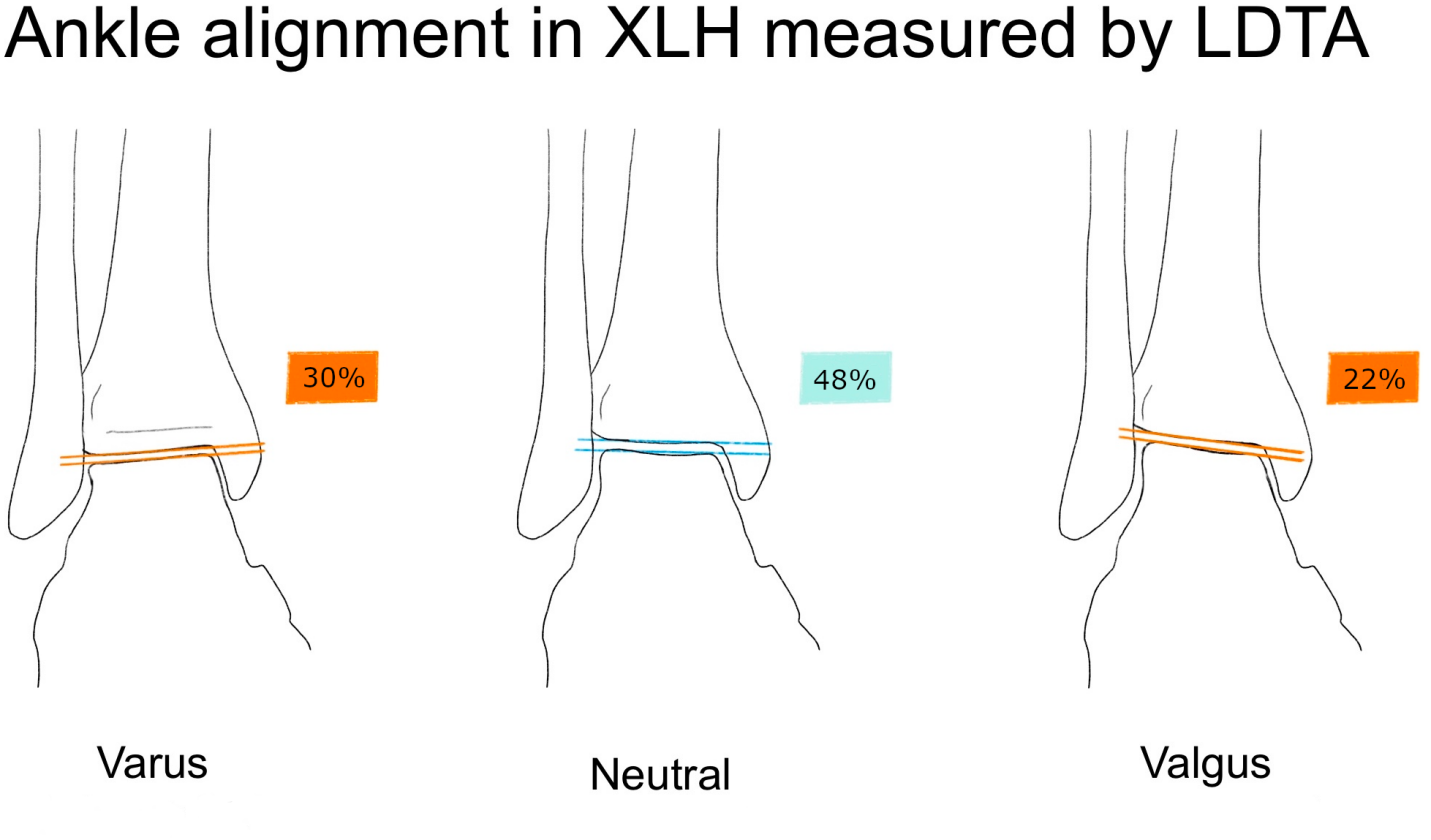
Illustration of the lateral distal tibial angle (LDTA) and ankle alignment in XLH patients (n = 46 limbs), norm values from Paley (11). Normal ankle alignment was defined as LDTA between 86 and 92 degrees, Varus malalignment was defined as LDTA > 92°, valgus malalignment as LDTA < 86° (11).

Ankle valgus deformity (**Figure 3**) was detected in 21.7 % and ankle varus deformity in 30.4 % of the limbs. Summary statistics are described in **Table 2**.

**Table 2:** Comparison for gait analysis data between XLH patients and the control group.

	XLH patients (n=34 limbs)				Control (n = 24 limbs)				
	Mean	SD	Min	Max	Mean	SD	Min	Max	P value
**Cadence (steps/min)**	109.6	7.9	92.3	122.2	115.9	8.5	99.1	138.6	**.006**
**Step width (m)**	0.112	0.042	0.039	0.183	0.080	0.022	0.026	0.149	**<.001**
**Walking speed normalized (m/s)**	1.0	0.196	0.510	1.3	1.3	0.133	0.946	1.8	**<.001**
**Ankle ROM (df/pf; deg)**	27.9	3.4	22.9	35.4	34.8	5.5	22.6	50.5	**<.001**
**Maximum ankle dorsiflexion (deg)**	16.3	3.5	9.0	23.4	14.5	3.3	6.1	21.7	**0.006**
**Ankle moment (df/pf; Nm/kg)**	1.2	0.3	0.4	1.7	1.6	0.135	1.2	2.0	**<.001**
**Ankle power maximal value stance phase (W/kg)**	2.5	0.9	0.7	4.2	3.9	0.642	2.0	6.0	**<.001**
**Gait Deviation Index**	69.7	17.4	40.8	101.1	100				**<.001**
**Gait Posture Score**	8.7	3.2	4.6	16.5	3.9	0.789	2.3	7.0	**<.001**

**XLH, X-linked hypophosphatemia; SD, standard deviation; Min, minimum; Max, maximum; deg, degrees. The p values are for comparison of study population and control group data. In the sagittal plane: positive values = anterior (ant)/dorsiflexion (df)/extension (ext) and negative values = posterior (post)/plantarflexion (pf)/flexion (flex).

### Osteoarthritis, enthesopathies and pseudofractures

**Figure 4:**
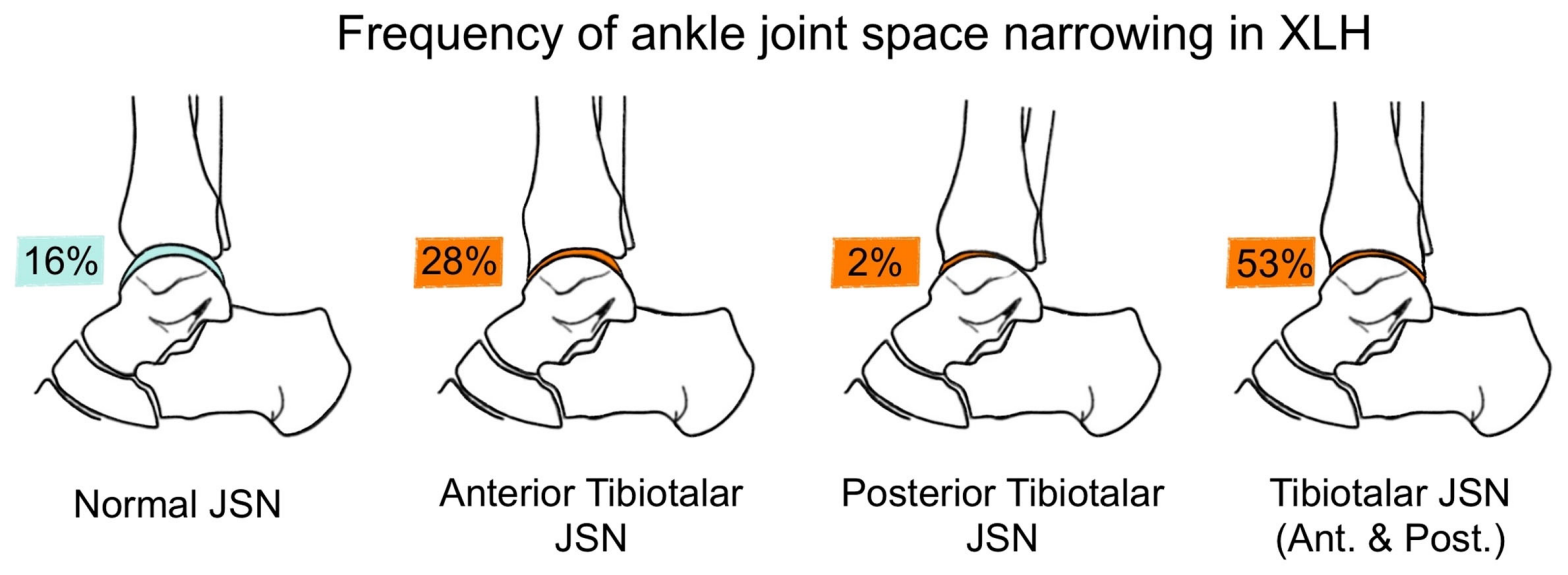
Frequency of mild to severe JSN and normal joint space in the tibiotalar joint in patients with XLH 34 limbs.

**Figure 5:**
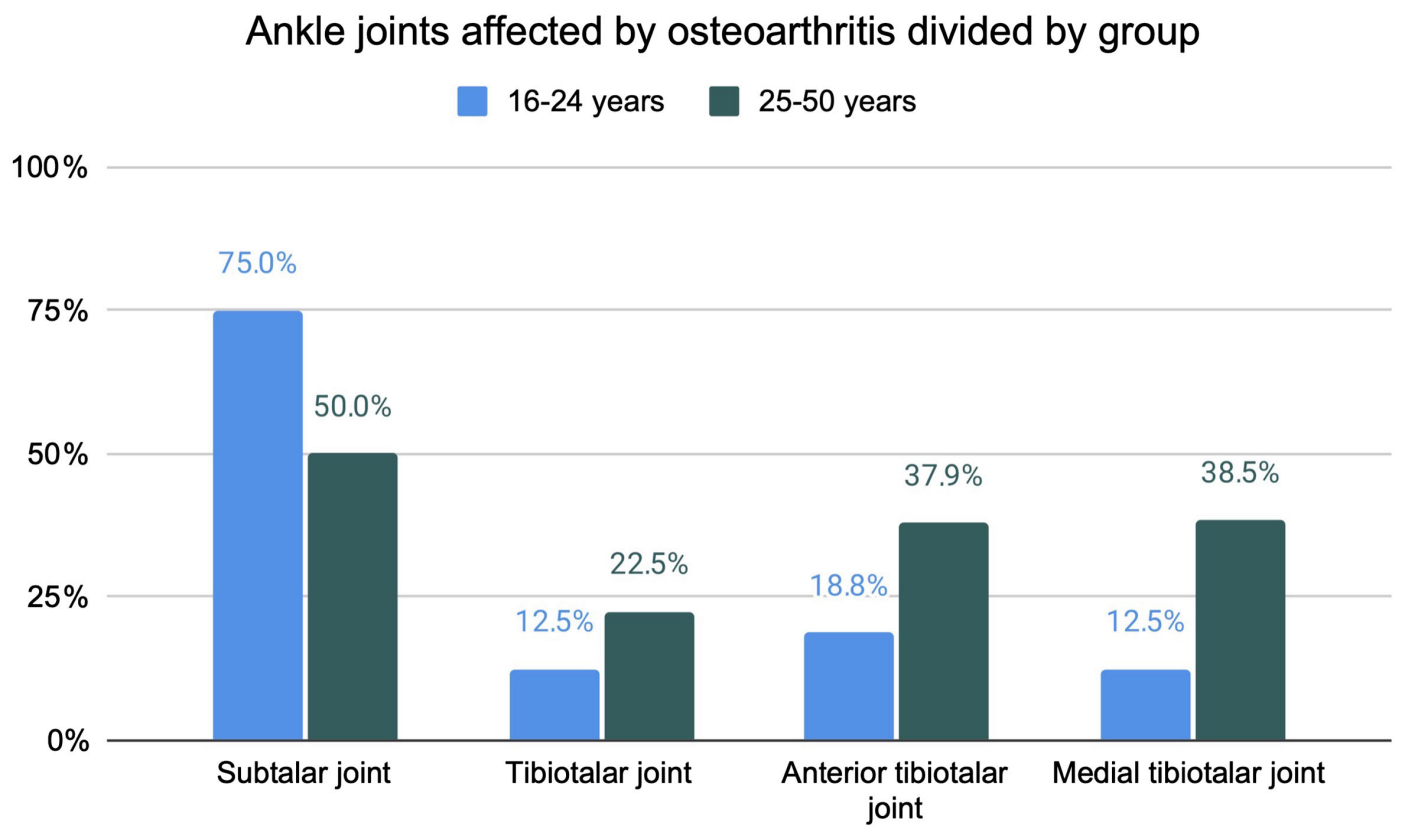
Frequency of ankle joints affected by moderate to severe JSN divided by age.

**Figure 6:**
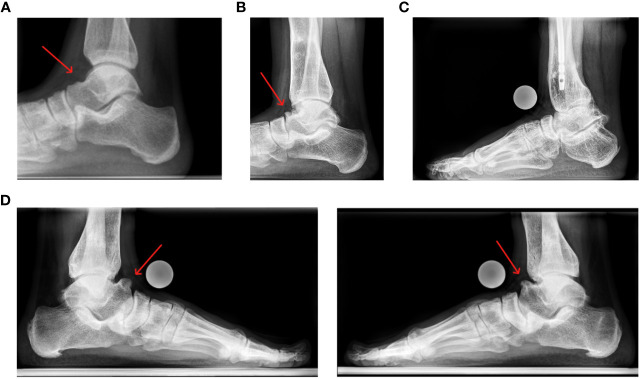
Lateral view of the ankle joint: **(A)** 21 year old female patient presenting with a minor anterior talar osteophyte development; **(B)** 19 year old female patient presenting a severe anterior talar osteophyte. **(C)** Lateral left ankle of a 32 year old male patient, state after numerous tibial and femoral osteotomies with OA and osteophytes of the tibia and talus. **(D)** lateral feet of a 30 year old male patient, state after multiple prior surgeries of both lower limbs. The patient is currently pain free but reports restricted ankle dorsiflexion.

The anterior tibiotalar (31.1%) and the subtalar joint (59.1%) were found to be a predilection site for moderate to severe JSN in XLH patients (**Figure 4**). Sub analysis of the patients split into two age groups (16 - 24y (n=16 legs), 25 - 50y (n = 30 legs)) revealed an increase of osteophytes and JSN with age (**Figures 5**, **6**).

The medial talus was found to be the most common site for osteophytes (81.6%) presence on ap radiographs while the medial tibiotalar joint, on the other hand, for JSN (69.0%).

Lateral radiographs revealed pronounced JSN and osteophyte development, especially in the anterior tibia (68.9%) and talus (77.8%). Moderate to large subtalar osteophytes were the most prevalent in the lateral radiographs and present in 34 (75.6%) ankles.

**Figure 7:**
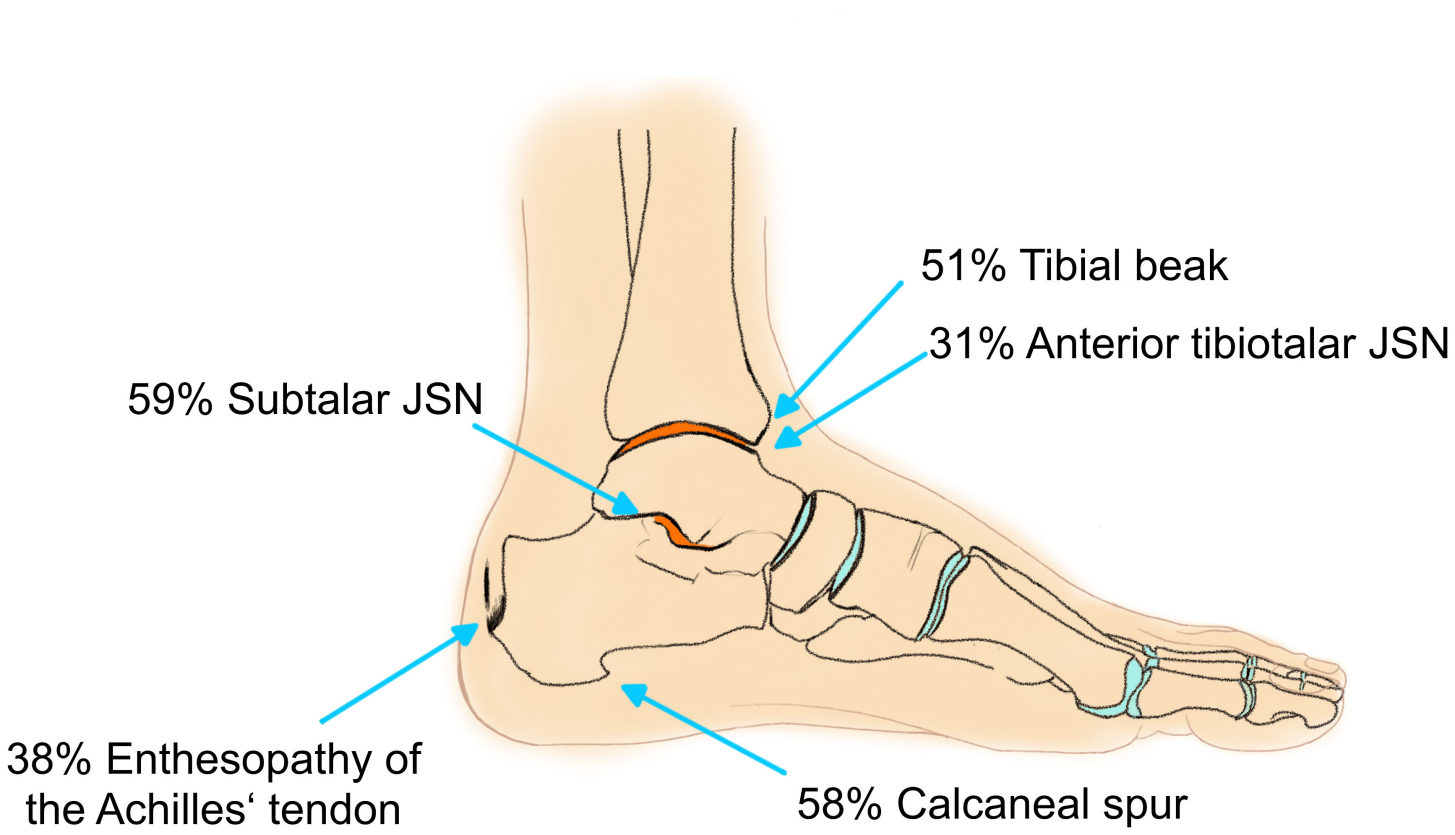
Frequency of enthesopathies and moderate to severe JSN of the ankle joint: orange surface indicates predilection sites for OA.

Enthesopathies of the ankle were common in XLH patients (**Figure 7**). Seventeen (37.8%) ankles presented with calcification around the insertion area of the achilles tendon, 24 with dorsal calcaneal spurs (53.3%) and 26 (57.8%) with a plantar calcaneal spur. Thirtyfive (79.5%) feet presented a marked processus posterior tali. A talar beak was present in 15 (33.3%) ankles while a tibial beak was found in 23 (51.1%) ankles.

Pseudofractures of the tibia and fibula on AP radiographs were only present in one 29 year old patient (2 of the tibia, 1 of the fibula). Ossification of the syndesmosis and/or of the membrana interossea was detected in 14 ankles (35.0%).

### Physical examination

XLH patients showed tight lateral and medial ligaments (inversion and eversion stress test) as well as tight anterior and posterior talus movement (Drawer test) (**Table 1**), however, subjective instability was reported in 27.3% of the limbs. 24.2% of XLH patients reported pressure pain in the achilles tendon, out of which five limbs (55.6%) also reported regular achillodynia.

Ankle dorsiflexion was significantly decreased in comparison to normal dorsiflexion angles in adults (p < 0.001). The foot distance from the wall in the weight bearing lunge test (WBLT) was significantly reduced in comparison to the reference norms (p < 0.001) and 51.5% measured out of the reference range (18). Average active ankle plantar flexion was not reduced in clinical examination.

Patients with XLH scored a reduced AOFAS (higher scores indicate no pathologies of the hindfoot): hindfoot: 86.1 ± 16.7 (range: 48 - 100); midfoot: 90.5 ± 14.8 (range: 43 - 100). One third of the participants experienced mild to moderate pain in the hindfoot (27.3%). 24.2% reported limited recreational and daily activities, reduced walking distance of 4 to 6 blocks (24.2%), moderate to marked restriction in inversion and eversion (27.3%) and severe difficulties when walking on uneven ground (33.4%). One third (35.3%) of the participants reported to be moderately physically active.

### Gait analysis

Kinetic and kinematic gait analysis data were compared between the XLH group and a historic control group of 88 healthy adults, obtained from our gait laboratory database. The mean age of the control group was 29 ± 8 years with the age ranging from 21 to 50 years. The mean height of the norm group was 161.8 ± 43.7 cm (range: 160.3 to 194 cm); mean weight 69.4 ± 12.6 kg (range: 47.2 to 101.7 kg); and average BMI 26.5 kg/m^2^.

**Figure 8:**
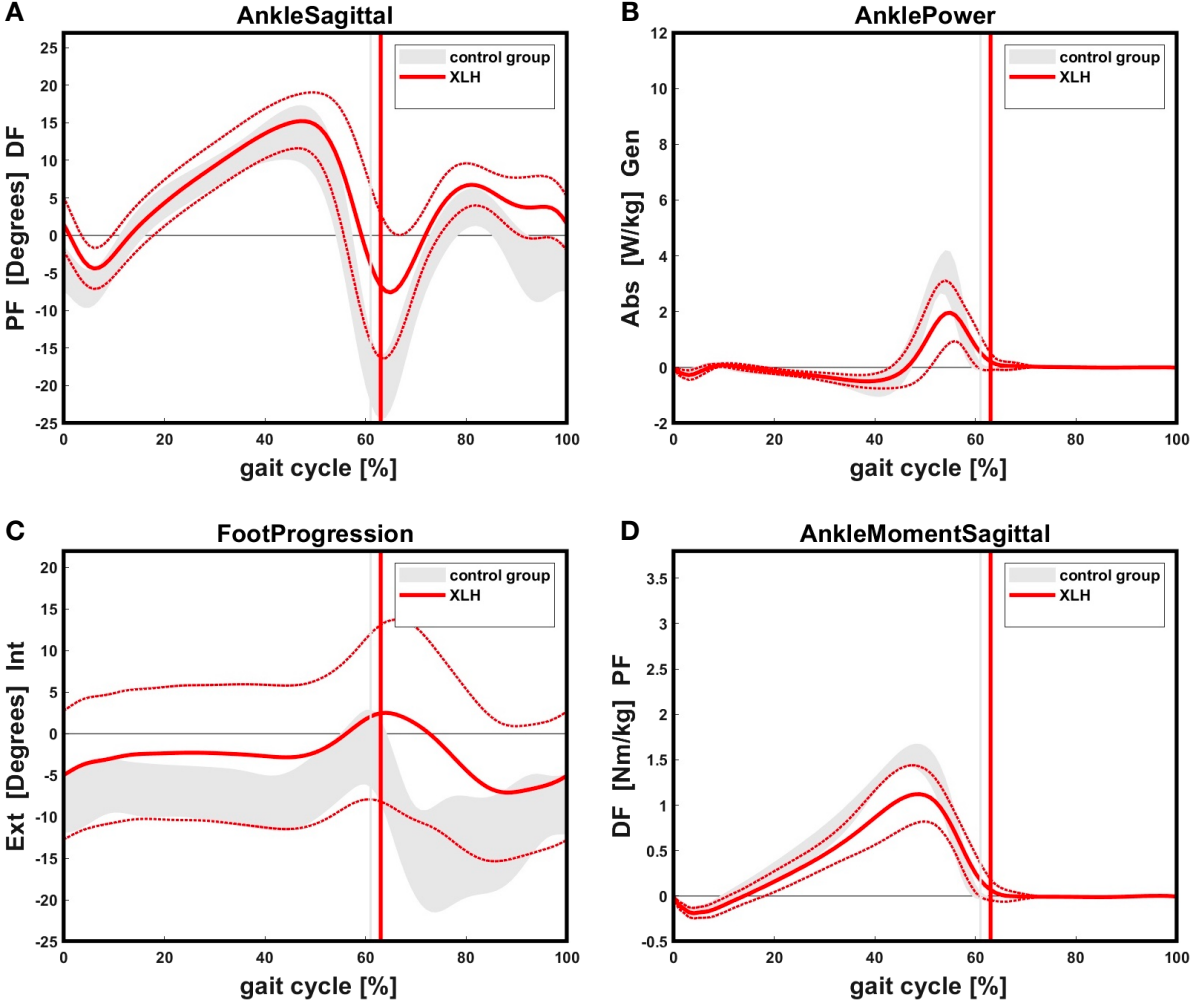
**(A)**: XLH patients showed reduced ankle plantar flexion in initial swing as well as in terminal swing an increased dorsiflexion compared to the control group. Solid (red) line indicates the XLH group (+ 1 SD), gray band indicates the control group (+ 1 SD). Positive values indicate dorsiflexion of the ankle, negative values indicate plantarflexion. **(B)**: XLH patients showed reduced ankle power during push-off phase compared to the control. **(C)**: XLH patients presented with increased internal foot progression during stance and swing phase compared to the norm. The standard deviation showed a high variability. Positive values indicate internal, negative values indicate external rotation of the foot. **(D)**: Patients with XLH showed reduced maximum sagittal ankle plantarflexion moment in the terminal stance phase.

Patients with XLH (n = 17) showed reduced ankle power, ankle plantar flexion moment and ankle plantar flexion, as well as increased internal foot progression compared to the norm group (p < 0.05) (**Figure 8**). Significantly slower walking speed (p = 0.003), a broader step width (p = 0.020), higher cadence (p < 0.001) and lower overall gait quality (gait deviation index (GDI)) (p < 0.001) were observed (**Table 2**).

### Quality of life

FAOS (higher scores indicate no pathologies): XLH patients scored the lowest test scores in the subcategories “activity in sports” (78.8 ± 27.8%; range: 20 - 100%); and “quality of life” (72.0 ± 26.6%; range: 12.5 - 100%)(n = 16).

SF36v2 (higher scores indicate no pathologies): Patients reported lower physical functioning (65.0 ± 23.1%; range: 25 - 100%), vitality (48.8 ± 25.1%; range: 10 - 85%), general health perception (56.3 ± 25.2%; range: 20 - 100%) and higher pain sensations (69.5 ± 23.1%; range: 22.5 - 100%) compared to the reference group (19). Emotional wellbeing and social functioning were similar to the reference group (19).

WOMAC (lower scores indicate no pathologies): XLH patients reported a mean pain sensation of 10.0 ± 12.2% (range: 0 to 42%) and an average 25.3 ± 28.3% (range: 0 to 85%) ankle stiffness. Moreover, XLH patients reported a mean loss of ankle function of 9.6 ± 13.5% (range: 0 to 48.2%). Overall XLH patients scored a mean WOMAC score of 11.0 ± 13.8%. In consideration of age and BMI, pre-obese or obese XLH patients aged 35 to 50 years reported worse sub scores in stiffness and function; pre-obese or obese patients of age group 25 to 34 reported worse test scores in all categories compared to the norm group (20).

### Gait parameter interactions

Reduced hindfoot function (AOFAS Score) had a significant impact on the GDI (F(2.27) = 5.857, p = 0.008). Patients reporting limited daily and recreational activities scored a significantly lower mean GDI (47.9 ± 7.7, p = 0.027) compared to patients with no limitations (74.8 ± 16.3).

**Figure 9:**
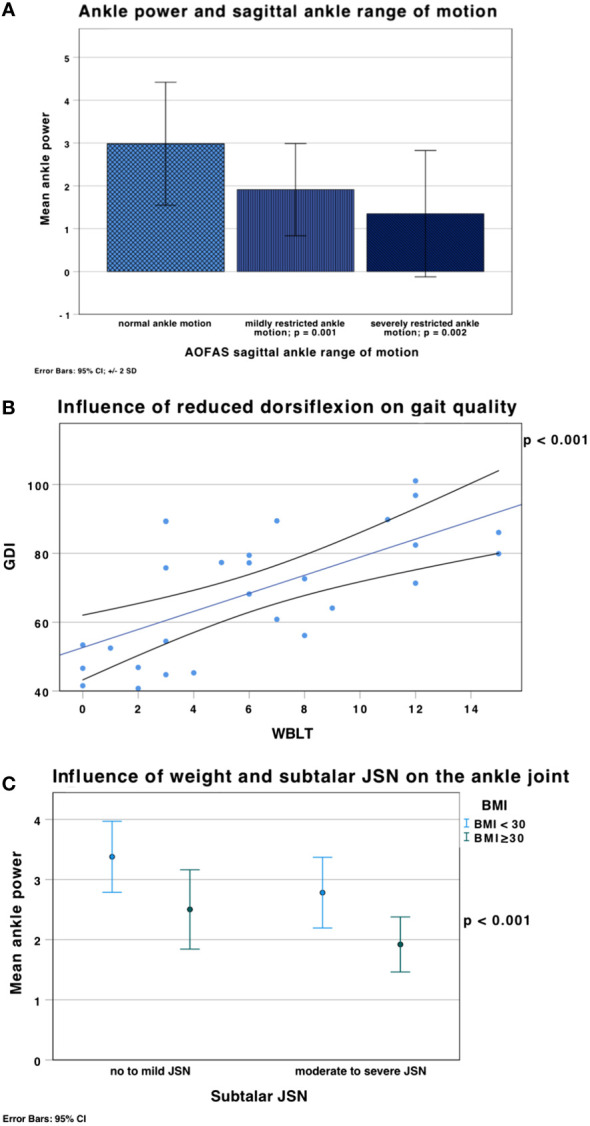
**(A)** Mean ankle power (error bars ± 2 SD) was significantly lower in ankles with mildly restricted sagittal ankle ROM in the AOFAS score (F(2,27) = 8.968, p = 0.001); ankle power was lower in patients with severely restricted ankle ROM (1.35 ± 0.74 W/kg; p = 0.002), which was defined as ROM < 15°, compared to normal (2.85 ± 0.8 W/kg) or mildly restricted ankle motion (1.9 ± 0.5 W/kg). **(B)** Linear correlation between the GDI and the WBLT (weighted ankle dorsiflexion). Reduced distance from the wall in the WBLT (= reduced ankle dorsiflexion) (x axis) led to reduced gait quality (lower GDI) (y axis) (p < 0.001). **(C)** Influence of weight and subtalar JSN on ankle power shown in mean ± SD. Obesity (green), defined as a BMI ≥ 30 kg/m^2^, a higher BMI is associated with lower ankle power (p < 0.001).

The GDI was lower for patients with self-reported severe ankle motion restriction (47 ± 6.9; p = 0.006) compared to normal or mild restriction. Furthermore, patients with reported moderate or severe hindfoot difficulties on uneven walking surfaces had a statistically significant lower gait quality (GDI: t(28) = 3.215; p = 0.003; gait posture score (GPS): t(28) = -2.341; p = 0.027) and ankle power (t(28) = 2.522; p = 0.018). Ankles with valgus malalignment also showed reduced mean ankle power (F(2,27) = 6.150; p = 0.006) (**Figure 9A**) and gait quality (GDI: F(2,27) = 3.613; p = 0.033) compared to ankles with normal alignment.

Tibial procurvatum led to significantly lower ankle power (t(28) = 2.824; p = 0.004).

Reduced active ankle dorsiflexion in the WBLT correlated with reduced ankle power (r = 0.52; p = 0.005) and gait quality: GDI (r = 0.66; p < 0.001); GPS (r = -0.43; p = 0.024) (**Figure 9B**).

XLH patients with a higher BMI presented with lower ankle power (r = -0.63; p < 0.001), gait quality (GDI: r = -0.51; p = 0.002; GPS: r = 0.57; p < 0.001) and higher incidence of moderate to severe subtalar JSN (**Figure 9C**).

Ankle power was also found to be significantly lowered in ankles with severe subtalar JSN compared to normal subtalar JSN (F(3,30) = 3.617, p = 0.023), as well as in ankles with moderate subtalar JSN compared to mild subtalar JSN (p = 0.037).

Subjective ankle instability was not associated with a higher frequency of malaligned ankles; and was found in 10.8 % of varus, 8.1 % valgus and 5.4 % neutral aligned limbs. Therefore, no correlation between ankle instability and alignment was found.

## Discussion

This study reports on details of foot and ankle function including radiographic, clinical, kinetic and QoL parameters in patients with XLH. The objective of this study was to assess ankle function and structural changes in patients with XLH compared to historic norm data. Furthermore, we hypothesized that impairment of ankle function leads to decrease of quality of life parameters in adolescents and adults with XLH. The main results are the following: Our XLH cohort presented with premature OA especially in the subtalar and anterior tibiotalar joints and enthesopathies of the calcaneus. Ankle ROM restrictions were observed in gait analysis, clinical examination as well as patient reported scores. Patients with XLH showed reduced ankle power during push-off. No lateral or medial ligament instability of the ankle joint was found in clinical examination. The AOFAS, SF36, FAOS and WOMAC scores showed a reduced mean physical and/or ankle function in patients with XLH.

Various studies reported reduced QoL as part of the burden of disease in patients with XLH, focusing on bone and joint pain as well as joint stiffness (2, 3). Ankle pain is the second most common site for joint pain in children, following knee pain, and accounts for third place of the lower limb in adults with XLH (2). Although ankle pain is frequently reported in studies, details on the pathomechanism, such as the role of enthesopathies on biomechanics, specific disease-associated disturbances of cartilage formation or possible impairments in muscle and tendon composition, and the impact on ankle function are still lacking. This study aimed to address possible specific co-factors as a leading cause for ankle OA development and functional impairment.

Our study revealed the tibiotalar joint as well the subtalar was highly affected by JSN. Ankles with subtalar JSN also presented the highest percentage of osteophytes and lower ankle power. Moderate and severe subtalar JSN as well as decreased ankle power was highly present in obese patients. It is important to mention that half of included ankles of adolescent and young adult XLH patients (16-24 years) presented with subtalar (moderate and severe) and tibiotalar (mild to severe) OA. This evidence suggests that a majority of XLH patients develop ankle OA at a younger age compared to the general population due to lifelong abnormal mechanical loading of the joints caused by skeletal deformities (6). However, in our opinion early OA development in XLH patients cannot be explained through mechanical factors alone. The disease presumably has an impact on cartilage degeneration and the development of ankle OA caused by a disease specific pathomechanism, as well as prior surgical interventions.

The importance of joint stiffness in patients with XLH as a limiting factor of everyday life as seen in reduced QoL, increased pain level and lower gait quality was documented in various studies (2–4), however, data focusing on the impact of the ankle joint on daily function is lacking.

XLH patients presented with a lower ankle ROM in the clinical examination and gait analysis. This correlates with similar results of other recent studies (6). The hindfoot was found to be more affected by pathologies than the midfoot (AOFAS score). Interestingly, active plantarflexion was not reduced in the clinical examination, even though plantarflexion was limited during gait. Steele et al. (6) reported gait analysis results of 9 adults with XLH and found a reduction of plantarflexion in gait analysis of more than half the normal range from terminal stance to pre-swing in patients with XLH compared to a healthy norm group. Furthermore, the reduced plantarflexion continued into the swing phase, achieving near-neutral dorsiflexion during mid-swing (6).

A high frequency of talar and subtalar osteophytes, enthesophytes at the insertion site of the achilles tendon and restricted plantar flexion of the ankle in initial and terminal swing phase was present in XLH patients. Enthesophytes of the achilles tendon and calcaneal enthesophytes may contribute to restricted ROM and pain.

A recent study observed development and progression of enthesopathies of the achilles tendon in Hyp mice as a mechanical adaptation to osteomalacia (21). While comparable data in humans with XLH is missing, gait analysis and further functional testing (including the muscle tendon complex) might be helpful in answering this clinically highly relevant research question.

Ankle instability has been described as an important factor for early onset of ankle OA (22). Therefore, lateral and medial ligaments of the ankle are of the utmost importance in the stabilization of the ankle joint and the prevention of ankle OA. This study found no correlation between varus malaligned ankles and ankle instability or OA under clinical examination by a specialized foot and ankle surgeon; all ankles were found to be stable with tight ligaments. However, every fourth patient reported subjective ankle instability which may be traced back to degenerative osseous processes and additional lower limb deformities; but not necessarily to ligament instability.

Ankle power and gait quality (GDI, GPS) were significantly influenced by decreased ankle ROM and lower AOFAS scores. These ankle specific findings emphasize that self-reported patient complaints are clinically highly relevant.

In children and adults with XLH a decreased GDI has been described (4, 5, 23). While our study cohort overlaps with the study population of Mindler et al. (4) we confirmed decreased GDI values and additionally observed reduced ankle function in adults with decreased gait quality. Therefore, the GDI seems to be a relevant parameter to partly reflect ankle function in adults with XLH.

A majority of XLH patients in this study cohort were considered pre-obese and obese compared to the Austrian mean BMI. Increased BMI was not only associated with lower gait quality scores in previous studies (4, 5), corresponding to findings of our study with an overlapping cohort (4), but also seems to be a highly relevant co-factor for functional ankle impairment for patients with XLH. Pre-obese or obese patients reported worse sub scores in regards to ankle stiffness and function on questionnaires.

Our study cohort showed increased internal tibial malrotation similar to recent reports on children and adults with XLH (4, 5, 23) associated with gait disturbances. Moreover, maltorsion of the tibia also leads to increased and abnormal intra-articular contact forces leading to accelerated arthritis development of the ankle due to the change of joint biomechanics (13). An internal or external malrotation of the tibia of 20° was found to increase peak pressure in the ankle joint (13).

Procurvatum deformity of the femur or tibia is another typical skeletal deviation in patients with XLH (4). Mindler et al. found reduced ankle and knee ROM in adults with XLH with increased anterior tibial bowing (4). Therefore, we hypothesized that the procurvatum deformity of the tibia contributes to decreased function of the ankle. In our study population tibial procurvatum deformity led to lower ankle power.

In our cohort, nearly all XLH patients reported to have undergone prior orthopedic surgery. Due to the design of this study, we were not able to determine whether preventive or OA induced surgical interventions on the ankle were undertaken.

Total knee and hip joint replacement has been effective in treating XLH adults with OA (24, 25). It has been reported that ankle arthroplasty in healthy patients with severe ankle OA improved sagittal ankle ROM (26). However, while this procedure might be an effective treatment for XLH patients, severe tibial maltorsion and the lack of knowledge on the pathomechanism of ankle pain in XLH can lead to difficulties that have to be addressed with further studies.

While fatigue, muscle weakness and chronic pain are common features of XLH (3), there is a considerable lack of pathomechanical data on a muscular influence on clinical symptoms in XLH. This study for the first time includes kinetic data of adolescents and adults with XLH. However, despite ankle power being significantly decreased, a muscular cause for power reduction could not be identified due to our study design. Further studies on musculoskeletal aspects in XLH, particularly on muscle function, are needed to adequately evaluate all factors contributing to known clinical features of XLH.

Overall, participants of this study reported lower QoL in all age groups, which is not surprising, considering OA with marginal osteophytes in the general population is the leading cause for pain and physical disability (27). Previous studies reported a lower vitality, role limitation due to emotional problems and social functioning in patients with XLH (2). However, in this cohort adolescents and adults with XLH reported a surprisingly good emotional wellbeing and general health perception. This could be partially explained with improving healthcare and a multidisciplinary setting including the option for psychological counseling for all age groups.

As a clinical consequence of our study we recommend, especially in cases of ankle pain even in young patients with XLH, to comprehensively analyze both the talocrural and subtalar joint using thorough clinical examination and radiographic analysis for osteoarthritis, enthesopathies and tibial deformities.

Although this study identified parameters for the development of ankle pain, it remains unclear whether osteoarthritis, tibial deformities, enthesopathies, reduced ROM, structural changes, or even bio-pathological pathways or a combination of the aforementioned are the main cause of pain in ankle joint complaints of patients with XLH. Further studies focusing on this highly relevant topic are needed to comprehensively assess the causes for joint pain in this rare bone disease.

### Limitations

The present study has several strengths, but also limitations. The study analyzed a retrospective cohort and additionally invited patients for a single time examination. While methodology and performing team for radiographic and gait analysis has been exactly the same for both data sets, a potential population bias regarding recruitment cannot be ruled out. We report on a homogenous study population, including genetically-confirmed, young XLH patients, to avoid a bias in gait deviations due to increased age. Furthermore, only four male participants were eligible for the study, therefore, gender specific differences could not be evaluated. Based on the heterogeneity of XLH our patient cohort had a varying frequency and severity of prior surgical procedures with some patients not having had surgery at all. This complicated the interpretation of surgical history.

OA of the subtalar joint might be overvalued in our study due to maltorsion and other XLH related deformities; further studies should evaluate through MRT or CT whether cartilage damage is truly this severe. An additional limitation might be missing radiographic values that could not be graded due to disease-specific maltorsion and deformity of the lower limb, as well as some missing patient data regarding weight and height. This data was taken from the gait analysis laboratory to ensure exact measurements and therefore only 17 patients had a complete record. They were defined as missing data. Furthermore, radiographic differentiation of osteoarthritis, osteophytes and enthesopathies can be difficult and it is plausible that it leads to a low inter-rater reliability.

The scores used in this study are validated (SF36, WOMAC, AOFAS), however they differ in reliability (28). To avoid another possible source of error, a goniometer was used to examine ankle ROM in the clinical examination. Lastly, this study conducted a very thorough analysis of the ankle joint, however pedobarography data was not available.

## Conclusion

Adolescent and adult XLH patients presented with increased premature ankle OA and enthesopathies. Ankle ROM restrictions were observed in gait analysis, clinical examination as well as patient reported scores contributing to lowered mean subjective ankle function and QoL on SF36, WOMAC and FAOS scores.

Patient reported outcome scores (lowered ankle function), clinical examination findings (decreased ankle ROM) and radiographic changes (OA of the ankle) highly correlated with reduced gait quality (GPS, GDI) and reduced ankle power in patients with XLH.

This study identified multiple parameters potentially leading to ankle pain in XLH making it crucial for orthopedic surgeons to adequately assess full ankle function and morphology in adolescents and adults with XLH prior to interventions.

Future studies should include the effect of long term Burosumab therapy, starting at an early age, on OA and enthesopathy development in adolescents with XLH. Moreover, more studies are needed to understand contributing co-factors and foot specific pathologies of XLH patients. A better comprehension of the lower limb pathology in XLH could help improve symptoms and target treatment options to increase quality of life and patient care in the future.

## Data availability statement

The raw data supporting the conclusions of this article will be made available by the authors, without undue reservation.

## Ethics statement

The studies involving human participants were reviewed and approved by Ethics Committee of the Vinzenz Group Vienna (EK 37/2020), Gumpendorferstraße 108 1060 Vienna, Austria. Written informed consent to participate in this study was provided by the participants’ legal guardian/next of kin.

## Author contributions

CA, FW-S, AS, AK, AR, RK, RG and GM contributed to conception and design of the study. CA and GM wrote the first draft of the manuscript. AS and AR wrote sections of the manuscript. CA, FW-S, GM did clinical data collection. AK did gait analysis data collection. CA and AK did statistical analysis. CA, AS, AR and GM performed data analysis. RK, AR, FW-S and RG did a review of the manuscript. GM organization of internal funding. All authors contributed to the article and approved the submitted version.
